# Longitudinal health-related quality of life in patients with pancreatic cancer stratified by treatment: a nationwide cohort study

**DOI:** 10.1016/j.eclinm.2024.103068

**Published:** 2025-01-21

**Authors:** Anne M. Gehrels, Pauline A.J. Vissers, Mirjam A.G. Sprangers, Nienke M. Fijnheer, Esther N. Pijnappel, Lydia G. van der Geest, Geert A. Cirkel, Judith de Vos-Geelen, Marjolein Y.V. Homs, Geert- Jan Creemers, Martijn W.J. Stommel, Lois A. Daamen, Marc G. Besselink, Johanna W. Wilmink, Hanneke W.M. van Laarhoven

**Affiliations:** a Department of Medical Oncology, Amsterdam UMC Location University of Amsterdam, Amsterdam, the Netherlands; b Cancer Center Amsterdam, Cancer Treatment and Quality of Life, Amsterdam, the Netherlands; c Department of Research and Development, Netherlands Comprehensive Cancer Organization (IKNL), Utrecht, the Netherlands; d Department of Surgery, RadboudUMC, Nijmegen, the Netherlands; e Department of Medical Psychology, Amsterdam UMC Location University of Amsterdam, Amsterdam, the Netherlands; f Department of Medical Oncology, Regional Academic Cancer Center Utrecht, Meander Medical Center Amersfoort, University Medical Center, Utrecht, the Netherlands; g Division of Medical Oncology, Department of Internal Medicine, Maastricht University Medical Center, Maastricht, the Netherlands; h Department of Medical Oncology, Erasmus MC, University Medical Center Rotterdam, Rotterdam, the Netherlands; i Department of Medical Oncology, Catharina Hospital, Eindhoven, the Netherlands; j Department of Surgery, Regional Academic Cancer Center Utrecht, University Medical Center Utrecht and St. Antonius Hospital Nieuwegein, the Netherlands; k Division of Imaging and Oncology, UMC Utrecht, Utrecht University, Utrecht, the Netherlands; l Amsterdam UMC Location University of Amsterdam, Department of Surgery, Amsterdam, the Netherlands

**Keywords:** Pancreatic cancer, Pancreatic adenocarcinoma, Health-related quality of life, Treatment strategies

## Abstract

**Background:**

Pancreatic adenocarcinoma (PAC) has a poor prognosis and substantially impairs health-related quality of life (HRQoL). Large studies on longitudinal HRQoL in patients with PAC, taking patient treatment into account, are lacking. This study aimed to investigate HRQoL over time in patients with PAC undergoing various treatments.

**Methods:**

This nationwide cohort study included patients diagnosed with PAC between 2015 and 2020. Data were collected from the Dutch Pancreatic Cancer Project (PACAP) and the Netherlands Cancer Registry. Patients were categorized into four groups based on treatment modality: resection (R-PAC), chemotherapy for localized disease (C-PAC), chemotherapy for metastatic disease (M1-C-PAC), and best supportive care (BSC). HRQoL was assessed using the EORTC QLQ-C30 and -PAN26 questionnaires at baseline, during treatment, and at 0–3 months and 3–6 months after treatment. Linear mixed models were used to analyze changes in HRQoL over time, with clinically relevant changes defined as a minimal mean difference of 10 points in absolute scores and reported with 95% confidence intervals.

**Findings:**

Overall, 1496 patients were included (673 [45.0%] female), of whom 675 (45.1%) in R-PAC, 319 (21.3%) in C-PAC, 340 patients (22.7%) in M1-C-PAC, and 162 (10.8%) in BSC group. In R-PAC, hepatic symptoms and health care satisfaction improved while role and social functioning deteriorated and eating related problems, side effects and fear of future health increased during treatment. In C-PAC, insomnia, pancreatic pain, hepatic symptoms decreased while diarrhea, side effects and fear of future health increased. In M1-C-PAC, pain, insomnia, pancreatic pain, hepatic symptoms, ascites and constipation decreased, sexuality improved while fear of future health and side effects increased. In BSC, hepatic symptoms decreased and flatulence increased.

**Interpretation:**

This nationwide study identified specific improvements and deteriorations in various HRQoL domains during 6 months follow-up. This information may be valuable in the clinical setting to inform patients on potential outcomes of the course of HRQoL during various treatment strategies.

**Funding:**

None.

**Research in context**

**Evidence before this study**

We conducted a PubMed search for relevant studies published between October 2017 and October 2022, using the terms “pancreatic cancer”, “quality of life”, and “health-related quality of life”. This search was updated in October 2024. Several studies have reported positive effects of various cancer treatments on patients' health-related quality of life (HRQoL), with the effects varying based on factors such as treatment type and disease stage. However, existing evidence primarily focuses on specific interventions or cross-sectional assessments, lacking a comprehensive, longitudinal perspective across all treatment strategies.

**Added value of this study**

This study addresses this gap by evaluating longitudinal HRQoL in 1496 patients with pancreatic cancer across various disease stages and treatment strategies, including surgery, systemic therapy, and best supportive care. By analyzing patient-reported outcome data, this research identifies distinct patters of improvement and deterioration in specific HRQoL domains over a six-month follow-up period.

**Implications of all the available evidence**

Comprehensive data on the potential outcomes of HRQoL during various treatment strategies enables clinicians to guide patient counseling and facilitate shared decision-making. Understanding the potential longitudinal HRQoL may better equip patients to make more informed decisions, ultimately improving their overall care experience in the context of this challenging diagnosis.

## Introduction

Pancreatic adenocarcinoma (PAC) is associated with a poor prognosis, characterized by a five-year overall survival of merely <5%.^1^ Although various treatment trajectories are available for PAC, they only offer limited gain in survival.^1^ It is thus highly important what the impact of these treatment strategies is on health-related quality of life (HRQoL), as any survival gain and alleviation of symptoms (benefits) must be weighed against treatment toxicity and deterioration in HRQoL (costs). In this context, it is essential to provide patients with sufficient information about the potential impact of these treatments on HRQoL, and to enable appropriate counseling during shared decision-making (SDM).^2^

A considerable amount of literature has been published on the association of distinct treatment regimens and patients' HRQoL. A recent study showed that patients with PAC in all disease stages experience a slight improvement in HRQoL after any form of cancer treatment in comparison with patients solely receiving best supportive care (BSC).^3^^,^^4^ In the curative setting, recent data suggest an initial decrease in HRQoL during the first phase following resection, followed by a subsequent improvement after 3–6 months,^5^, ^6^, ^7^, ^8^ with long-term survivors reporting an excellent quality of life.^9^^,^^10^ Furthermore, the impact of surgical procedures on HRQoL has been found to depend on the specific type of resection required and the chosen surgical approach.^5^^,^^11^

Although systemic treatment may induce toxicity, data from clinical studies have shown that first-line chemotherapy may improve or maintain HRQoL in patients with metastatic PAC.^12^^,^^13^ Additionally, the type of chemotherapy has been shown to influence HRQoL.^14^ For instance, in comparison to gemcitabine, FOLFIRINOX (fluorouracil, irinotecan, oxaliplatin) has been found to significantly improve HRQoL in patients with metastatic PAC.^15^ Another study evaluating the use of FOLFIRINOX in patients with locally advanced PAC, found that patients reported an improved HRQoL following treatment, despite a high toxicity rate.^16^ A study comparing gemcitabine plus nab-paclitaxel and gemcitabine alone in patients with advanced PAC found an increased survival in the combination group without increasing toxicity and deterioration of HRQoL.^17^

While separate studies have reported positive effects of various cancer treatments on patients' HRQoL, no study has reported on longitudinal HRQoL in patients with PAC undergoing different treatment strategies, including best supportive care. The aim of this study is to investigate the longitudinal HRQoL of patients across all stages of PAC who have undergone surgical treatment, systemic treatment or BSC.

## Methods

### Study design

This study comprises a nationwide, retrospective cohort. Patients diagnosed with PAC at any stage who participated in the Dutch Pancreatic Cancer Project (PACAP) between January 1, 2015 and December 31, 2021 were included. Additional clinical data were obtained from the Netherlands Cancer Registry (NCR).

### Data collection

NCR data consisted of patient (i.e., sex, age, WHO performance status (PS), comorbidities, time from diagnosis to death), tumor (i.e., location, TNM stage), and treatment characteristics (i.e., type of resection, systemic treatment and radiotherapy). The Charlson Comorbidity Index (CCI) was categorized as 0, 1 or ≥2 comorbidities. Primary tumor location was classified as head, body, tail or other/non specified, according to the ICD-O-3 guidelines. Tumor stage was based on clinical tumor-node-metastasis classification at the time of registration (UICC 7th edition during 2015–2016, 8th edition during 2017–2020).^18^^,^^19^ Patients were divided into four groups at baseline: 1) resection (either with or without (neo)adjuvant therapy) for localized PAC (R-PAC), 2) chemotherapy only for localized PAC (C-PAC), 3) chemotherapy for metastatic PAC (M1-C-PAC), and 4) BSC.

HRQoL data was prospectively obtained in the PACAP study, i.e., at inclusion and every three months during the first year of follow-up, and every six months during the second year of follow-up. In this study, assessment moments were reconstructed into baseline (i.e., before treatment), during treatment and 0–3 and 3–6 months after treatment (T0, T1, T2, and T3, respectively) for patients who received treatment. Baseline was defined as the assessment between date of diagnosis and start of the first treatment. If multiple questionnaires were completed during treatment, one was randomly selected. For the BSC group, assessment moments were reconstructed into 0–3 months after diagnosis and 3–6 months after diagnosis (T0 and T1 respectively).

HRQoL was assessed with the European Organisation for Research and Treatment of Cancer (EORTC) cancer-specific QLQ-C30 and pancreas-specific QLQ-PAN26.^20^^,^^21^ The QLQ-C30 consists of 30 items that are combined to form a global health status scale (GHS), five functioning scales (i.e., physical, role, emotional, cognitive, and social functioning), three symptom scales (i.e., fatigue, nausea and vomiting, pain), six individual items assessing symptoms (i.e., dyspnea, insomnia, appetite loss, constipation, diarrhea, and financial difficulties). The QLQ-PAN26 consists of 26 items that are combined to assesses nine disease symptoms and treatment side-effects (i.e., pancreatic pain, eating related problems, altered bowel habit, hepatic symptoms, side effects, cachexia, indigestion, ascites, flatulence) and five emotional scales/items specific to PAC (i.e., body image, healthcare satisfaction, sexuality, fear of future health, ability to plan the future). The scoring of the questionnaires was performed according to the respective manuals provided by the EORTC. For both questionnaires, the transformed scores range from 0 to 100. A higher score for the GHS represents better HRQoL and a higher score for functioning or emotional scales/items represents a higher level of functioning (i.e., better HRQoL), whereas a higher score for symptom scales/items represents a higher level of symptomatology, and hence poorer HRQoL.

### Statistical methods

Baseline characteristics were obtained using descriptive statistics. Categorical variables were presented as numbers and percentages. Continuous data were presented as median values with interquartile ranges (IQR). Unadjusted linear mixed model analyses, accounting for missing data at random, were performed to describe and assess trends in HRQoL over time. The dependent variable was one of the scale scores from the questionnaires, with time as a fixed effect to capture differences across measurement time points. Baseline scores were included as a reference point within the model to facilitate comparison of each subsequent time point to baseline values. A random intercept for each participant was included to account for baseline differences between individuals. Covariance structures were selected based on the lowest Akaike Information Criterion (AIC). The model was fitted using Restricted Maximum Likelihood (REML) estimation. QLQ-C30 and PAN26 scores were expressed as means with standard error (SE). Clinically relevant changes were defined as a minimal mean difference (MD) of 10 points in absolute scores between the score at a certain time point compared to baseline value (T0), based on the method of Osoba (1998),^22^ and were reported with 95% confidence intervals (CI). All analyses were performed using IBM SPSS Statistics (version 28).

### Ethics

Patients provided written informed consent for participation in the PACAP project and data linkage. The study proposal was approved by the privacy board of the NCR and the scientific committee of the Dutch Pancreatic Cancer Group,^23^ medical ethical approval was not required. This study was designed in accordance with the Strengthening the Reporting of Observational Studies in Epidemiology (STROBE) guidelines.^24^

### Role of funding

This study received no funding.

## Results

### Patient enrollment

**Fig. 1:**
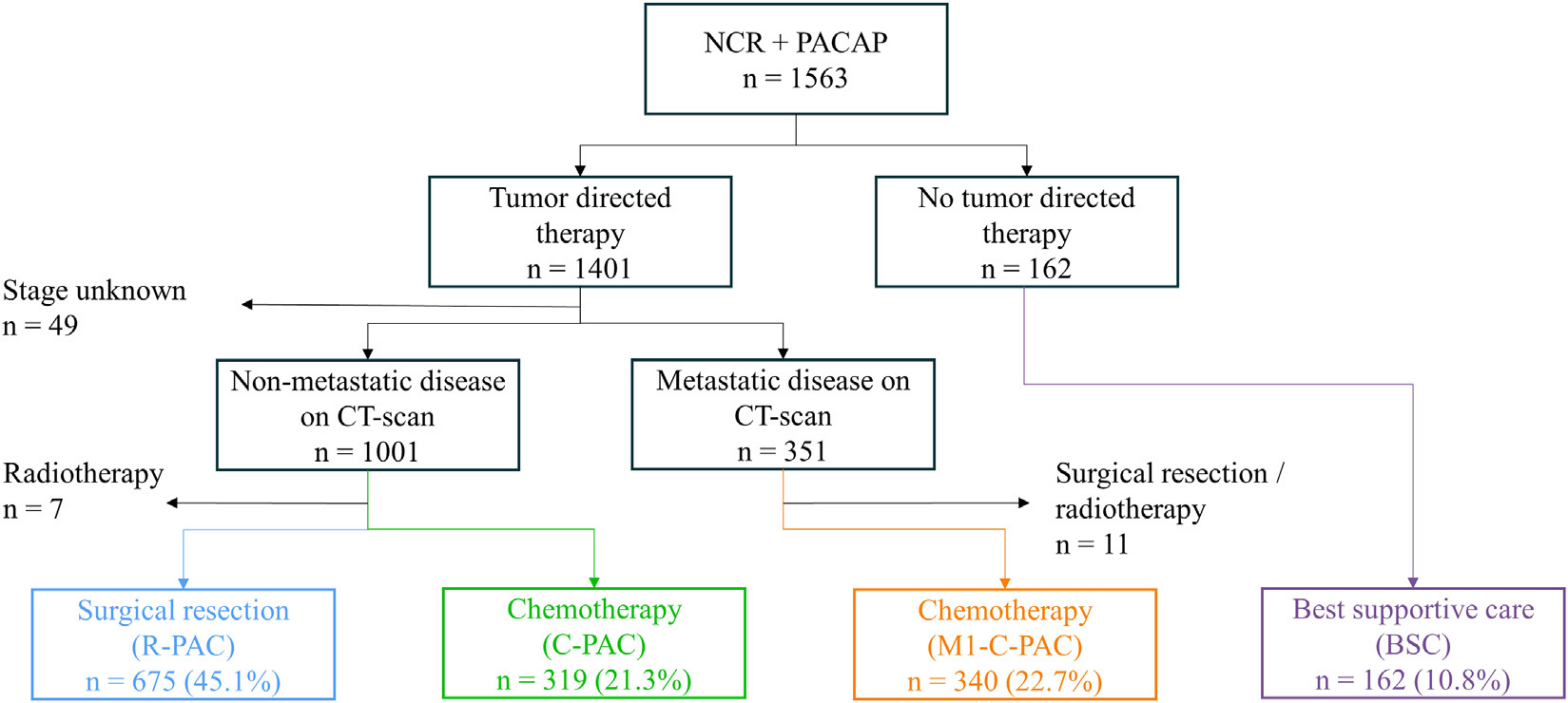
Patient inclusion.

Patient enrollment is schematically presented in Fig. 1. In total, 1563 patients were included. Patients with unknown clinical tumor stage (n = 49), who received radiotherapy only (n = 7) or who received surgical resection or radiotherapy but had metastatic disease (n = 11) were excluded. Of the 1496 eligible patients, 675 patients (45.1%) were in the R-PAC, 319 patients (21.3%) in the C-PAC, 340 patients (23.7%) in the M1-C-PAC, and 162 patients (10.8%) in the BSC group.

### Patient, tumor and treatment characteristics

In the total cohort, 673 (45.0%) patients were female. Median age was 67 (IQR: 60–73) years. More than half of the patients (51.1%) had a CCI of 0 and most patients had WHO PS of 0 (41.0%). Mortality rates increased progressively with time in each group after completing the last questionnaire. Patient, tumor and treatment characteristics of the subgroups are shown in Table 1.

**Table 1:** Patient, tumor and treatment characteristics.

	Total (n = 1496)	R-PAC (n = 675)	C-PAC (n = 319)	M1-C-PAC (n = 340)	BSC (n = 162)
**Patient characteristics**					
*Sex (n, %)*					
Female	673 (45.0)	303 (44.9)	142 (44.5)	149 (43.8)	79 (48.8)
*Age, years, median (IQR)*	67 (60–73)	66 (60–73)	66 (60–72)	64.5 (57–70)	72 (66–78)
*CCI (n, %)*					
0 comorbities	765 (51.1)	320 (47.4)	171 (53.6)	189 (55.6)	85 (52.2)
1 comorbidity	440 (29.4)	209 (31.0)	92 (28.8)	94 (27.6)	45 (27.8)
≥2 comorbidities	233 (15.6)	119 (17.6)	45 (14.1)	44 (12.9)	25 (15.4)
Missing	58 (3.9)	27 (4.0)	11 (3.4)	13 (3.8)	7 (4.3)
*WHO performance status (n, %)*					
WHO 0	614 (41.0)	296 (43.9)	150 (47.0)	124 (36.5)	44 (27.2)
WHO 1	563 (37.6)	231 (34.2)	124 (38.9)	161 (47.4)	47 (29.0)
WHO 2–4	121 (8.1)	38 (5.6)	19 (6.0)	26 (7.6)	38 (23.5)
Unknown	198 (13.2)	110 (16.3)	26 (8.1)	29 (9.5)	33 (20.4)
Deceased within					
3 months after treatment		43 (6.3)	47 (14.7)	105 (30.9)	
6 months after treatment		111 (16.2)	131 (41.1)	203 (59.7)	
3 months after diagnosis					44 (27.2)
6 months after diagnosis					96 (59.3)
Missing		2 (0.3)	2 (0.6)	2 (0.6)	0
**Tumor characteristics**					
*Tumor location (n, %)*					
Head of pancreas	986 (65.9)	523 (77.5)	209 (65.5)	156 (45.9)	98 (60.5)
Body of pancreas	210 (14.0)	41 (6.1)	65 (20.4)	73 (21.5)	31 (19.1)
Tail of pancreas	189 (12.6)	84 (12.4)	15 (4.7)	71 (20.9)	19 (11.7)
Other	111 (7.4)	27 (4.0)	30 (9.4)	40 (11.8)	14 (8.6)
*Stage (n, %)*					
0/IA/IB	465 (31.1)	404 (59.9)	43 (13.5)		18 (11.1)
IIA/IIB	226 (15.1)	170 (25.2)	42 (13.2)		14 (8.6)
III	374 (25.0)	101 (15.0)	234 (73.4)		39 (24.1)
IV	431 (28.8)			340 (100)	91 (56.2)
*Number of metastatic sites (n, %)*^a^					
None	1033 (69.1)	658 (97.5)	305 (95.6)	1 (0.3)	69 (42.6)
One	319 (21.3)	17 (2.5)	13 (4.1)	220 (64.7)	69 (42.6)
Two or more	144 (9.6)		1 (0.3)	119 (35.0)	24 (14.8)
**Treatment characteristics**					
*Surgical resection (n, %)*					
Pancreatoduodenectomy		534 (79.1)			
Distal pancreatectomy		119 (17.6)			
Other		22 (3.3)			
*Chemotherapy (n, %)*					
No	248 (16.6)	86 (12.7)			162 (100)
Neoadjuvant only	186 (12.4)	186 (27.6)			
Adjuvant only	250 (16.7)	250 (37.0)			
Neoadjuvant and adjuvant	153 (10.2)	153 (22.7)			
Yes, no resection	659 (44.1)		319 (100)	340 (100)	
*Type of first-line chemotherapy (n, %)*					
FOLFIRI(NOX)	850 (68.1)	316 (53.7)	258 (80.9)	276 (81.2)	
Gemcitabine based	395 (31.7)	273 (46.3)	60 (18.8)	62 (18.2)	
Missing	3 (0.2)		1 (0.3)	2 (0.6)	
*Radiotherapy (n, %)*					
No	1270 (84.9)	549 (81.3)	231 (72.4)	328 (96.5)	
Yes, neoadjuvant	126 (8.4)	126 (18.7)			
Yes, no resection	100 (6.7)		88 (27.6)	12 (3.5)	

R-PAC, resection (either with or without (neo)adjuvant therapy) for localized PAC; C-PAC, chemotherapy only for localized PAC; M1-C-PAC, chemotherapy for metastatic PAC; BSC, best supportive care; CCI, Charlson Comorbidity Index; FOLFIRI, Irinotecan, 5-Fluorouracil; FOLFIRINOX, Oxaliplatin, Irinotecan, 5-Fluorouracil.

a Metastases could have been found after start of neoadjuvant treatment.

### HRQoL of patients with localized PAC who underwent a resection

**Fig. 2:**
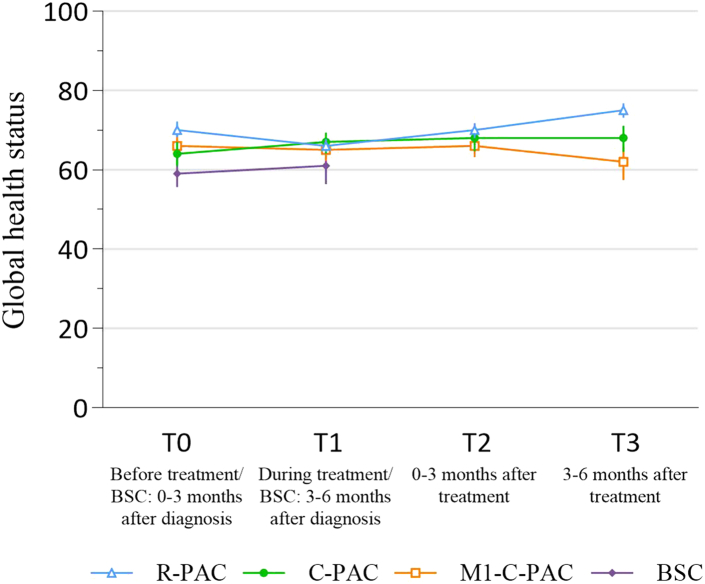
Global health status over time of patients with localized PAC who underwent resection (R-PAC) or chemotherapy only (C-PAC) and patients with metastatic PAC who received chemotherapy (M1-C-PAC) or patients who received best supportive care (BSC).

In the R-PAC group (Table 2), the mean GHS at baseline was 70.2 (SE 1.0) and decreased during treatment (T1) (MD: −4.0, 95% CI [−6.2, −1.7]) and increased 3–6 months after treatment (T3) (MD: 4.8, 95% CI [2.4, 7.2]), although these changes were not considered clinically relevant (Fig. 2). Role functioning decreased clinically relevantly at T1 (MD: −14.5, 95% CI [−17.8, −11.1]) and T2 (MD: −10.1, 95% CI [−13.8, −6.4]). Social functioning deteriorated clinically relevantly at T1 (MD: −10.6, 95% CI [−13.5, −7.8]). Appetite loss decreased clinically relevantly at T3 (MD: −13.5, 95% CI [−17.2, −9.7]).

**Table 2:** EORTC-QLQ-C30 and PAN26 subscales of patients with localized PAC who underwent a resection (either with or without (neo)adjuvant therapy) (R-PAC).

	T0 | Before treatment (n = 312)	T1 | During treatment (n = 511)			T2 | 0–3 months after treatment (n = 458)			T3 | 3–6 months after treatment (n = 375)		
	Mean (SE)	Mean (SE)	Δ	95% CI	Mean (SE)	Δ	95% CI	Mean (SE)	Δ	95% CI
**QLQ-C30**										
Global health status	70.2 (1.0)	66.2 (0.8)	−4.0	−6.2, −1.7	69.7 (0.9)	−0.5	−2.7, 1.8	75.0 (0.9)	4.8	2.4, 7.2
Physical functioning	82.3 (1.0)	74.0 (0.8)	−8.3	−10.3, −6.2	75.5 (0.9)	−6.8	−9.0, −4.6	80.0 (0.9)	−1.3	−3.3, 0.7
Role functioning	74.2 (1.5)	59.7 (1.3)	**−14.5**	−17.8, −11.1	64.1 (1.4)	**−10.1**	−13.8, −6.4	74.1 (1.3)	−0.1	−3.6, 3.5
Emotional functioning	75.3 (1.1)	78.8 (0.9)	3.5	1.3, 5.6	81.3 (0.9)	6.0	3.7, 8.2	81.7 (0.9)	6.4	4.0, 8.7
Cognitive functioning	86.2 (0.9)	82.1 (0.8)	−4.1	−6.1, −2.1	82.1 (0.9)	−4.1	−6.2, −2.1	83.7 (0.9)	−2.5	−4.7, −0.4
Social functioning	81.1 (1.3)	70.5 (1.1)	**−10.6**	−13.5, −7.8	74.2 (1.2)	−6.9	−10.0, −3.8	81.4 (1.1)	0.3	−2.8, 3.3
Fatigue	33.7 (1.3)	43.5 (1.1)	9.8	6.8, 12.7	38.7 (1.1)	5.0	1.8, 8.0	30.9 (1.1)	−2.8	−6.0, 0.2
Nausea and vomiting	11.2 (1.0)	16.8 (0.9)	5.6	3.1, 8.1	9.4 (0.8)	−1.8	−4.3, 0.8	6.3 (0.7)	−4.9	−7.2, −2.6
Pain	21.9 (1.3)	21.8 (1.1)	−0.1	−3.0, 2.7	21.8 (1.2)	−0.1	−3.3, 3.0	18.4 (1.1)	−3.5	−6.6, −0.6
Dyspnea	10.9 (1.0)	15.7 (1.0)	4.8	2.4, 7.2	13.6 (1.0)	2.7	0.1, 5.2	10.4 (1.0)	−0.5	−3.0, 1.9
Insomnia	31.1 (1.7)	24.9 (1.2)	−6.2	−9.8, −2.6	25.5 (1.4)	−5.6	−9.5, −1.6	23.5 (1.3)	−7.6	−11.4, −3.7
Appetite loss	25.1 (1.6)	30.2 (1.3)	5.1	1.4, 8.9	21.1 (1.4)	−4.0	−7.9, 0	11.6 (1.1)	**−13.5**	−17.2, −9.7
Constipation	10.1 (1.1)	11.5 (0.9)	1.4	−1.4, 4.1	6.8 (0.8)	−3.3	−5.6, −0.8	6.1 (0.8)	−4.0	−6.6, −1.6
Diarrhea	13.3 (1.2)	19.2 (1.2)	5.9	2.9, 9.0	20.4 (1.2)	7.1	3.9, 10.4	16.6 (1.2)	3.3	0.2, 6.5
Financial difficulties	3.9 (0.8)	6.0 (0.7)	2.1	1.0, 3.5	7.0 (0.7)	3.1	1.8, 5.1	7.8 (0.8)	3.9	2.1, 5.8
**QLQ-PAN26**										
Body image	18.2 (1.1)	24.7 (1.1)	6.5	3.7, 9.2	23.9 (1.2)	5.7	2.6, 8.7	18.6 (1.2)	0.4	−2.5, 3.5
Pancreatic pain	24.4 (1.1)	21.7 (0.9)	−2.7	−5.2, −0.2	21.5 (0.9)	−2.9	−5.4, −0.3	19.7 (0.9)	−4.7	−7.2, −2.3
Eating related items	18.9 (1.4)	29.5 (1.2)	**10.6**	7.4, 14.0	22.6 (1.3)	3.7	0.3, 7.1	14.2 (1.1)	−4.7	−7.7, −1.6
Altered bowel habit	29.5 (1.5)	34.0 (1.1)	4.5	1.2, 7.8	36.7 (1.1)	7.2	3.8, 10.6	34.0 (1.3)	4.5	0.9, 8.1
Hepatic symptoms	26.4 (1.8)	5.4 (0.6)	**−21.0**	−24.7, −17.4	5.7 (0.6)	**−20.7**	−24.4, −17.1	5.8 (0.6)	**−20.6**	−24.3, −16.9
Health care satisfaction	32.5 (1.7)	36.2 (1.3)	3.7	−0.1, 7.5	45.7 (1.5)	**13.2**	9.0, 17.4	50.7 (1.7)	**18.2**	13.7, 22.7
Sexuality	46.2 (2.0)	53.4 (1.6)	7.2	2.9, 11.4	46.3 (1.7)	0.1	−4.3, 4.4	43.0 (1.9)	−3.2	−7.9, 1.4
Side effects	19.6 (1.1)	37.2 (1.0)	**17.6**	15.0, 20.3	29.4 (1.0)	9.8	7.3, 12.5	21.2 (0.9)	1.6	−0.9, 4.2
Cachexia	23.1 (1.3)	27.6 (1.0)	4.5	1.7, 7.4	26.3 (1.1)	3.2	0.3, 6.2	19.4 (1.1)	−3.7	−6.7, −0.6
Indigestion	18.6 (1.4)	26.6 (1.3)	8.0	4.6, 11.4	23.5 (1.3)	4.9	1.6, 8.2	20.6 (1.2)	2.0	−1.2, 5.0
Ascites	25.0 (1.5)	26.1 (1.2)	1.1	−0.23, 4.4	23.7 (1.3)	−1.3	−5.0, 2.3	23.3 (1.2)	−1.7	−5.4, 2.0
Flatulence	31.3 (1.5)	39.8 (1.2)	8.5	5.0, 11.9	40.3 (1.3)	9.0	5.5, 12.5	36.9 (1.4)	5.6	1.9, 9.4
Fear of future health	53.7 (1.5)	43.0 (1.2)	**−10.7**	−14.0, −7.5	42.3 (1.3)	**−11.4**	−15.0, −7.9	40.7 (1.4)	**−13.0**	−16.8, −9.2
Ability to plan the future	35.0 (1.7)	43.6 (1.4)	8.6	4.7, 12.5	34.2 (1.5)	−0.8	−4.9, 3.4	25.2 (1.5)	−9.8	−13.9, −5.6

Linear mixed model analysis at baseline (T0), during treatment (T1), 0–3 months after treatment (T2), and 3–6 months after treatment (T3). The delta (Δ) indicates the mean difference between the score at that time point compared to baseline value. SE, standard error; 95% CI, 95% confidence interval; n, number of patients who answered the specific question in the questionnaires. Higher mean scores indicate better quality of life for the functional scales, or more symptomatology (poorer quality of life) for the symptom scales.

Bold numbers indicate a clinically relevant difference.

Of the PAN26 subscales, hepatic symptoms decreased clinically relevantly (MD: −21.0, 95% CI [−24.7, −17.4]) and remained stable at 0–3 months after treatment (T2) and T3. Health care satisfaction improved clinically relevantly at T2 (MD: 13.2, 95% CI [9.0, 17.4]) and T3 (MD: 18.2, 95% CI [13.7, 22.7]). Eating related problems and side effects increased clinically relevantly at T1 (MD: 10.6, 95% CI [7.4, 14.0] and MD: 17.6, 95% CI [15.0, 20.3] respectively). Fear of future health deteriorated clinically relevantly at T1 (MD: −10.7, 95% CI [−14.0, −7.5]), T2 (MD: −11.4, 95% CI [−15.0, −7.9]), and T3 (MD: −13.0, 95% CI [−16.8, −9.2]).

### HRQoL of patients with localized PAC who received chemotherapy

In the C-PAC group (Table 3), the mean GHS at baseline was 64.0 (SE: 1.5) and no statistically or clinically relevant changes were observed over time (Fig. 2). Of the QLQ-C30, insomnia decreased clinically relevantly at T1 (MD: −11.8, 95% CI [−18.0, −5.5]), T2 (MD: −15.3, 95% CI [−21.5, −9.1]), and T3 (MD: −13.0, 95% CI [−19.3, −6.6]). Diarrhea clinically relevantly increased at T1 (MD: 10.9, 95% CI [5.6, 16.3]).

**Table 3:** EORTC-QLQ-C30 and PAN26 subscales of patients with localized PAC who received chemotherapy only (C-PAC).

	T0 | Before treatment (n = 148)	T1 | During treatment (n = 196)			T2 | 0–3 months after treatment (n = 166)			T3 | 3–6 months after treatment (n = 118)		
	Mean (SE)	Mean (SE)	Δ	95% CI	Mean (SE)	Δ	95% CI	Mean (SE)	Δ	95% CI
**QLQ-C30**										
Global health status	64.0 (1.5)	66.8 (1.4)	2.8	−0.8, 6.4	67.6 (1.5)	3.6	−0.1, 7.3	67.8 (1.7)	3.8	−0.3, 7.9
Physical functioning	77.9 (1.5)	76.4 (1.3)	−1.5	−5.2, 2.2	74.6 (1.4)	−3.3	−6.7, 0	75.0 (1.6)	−2.9	−6.8, 1.1
Role functioning	65.9 (2.5)	63.5 (2.0)	−2.4	−8.6, 3.8	66.5 (2.1)	0.6	−5.5, 6.6	67.5 (2.2)	1.6	−4.7, 7.8
Emotional functioning	71.4 (1.6)	79.9 (1.2)	8.5	4.9, 12.1	78.3 (1.3)	6.9	3.3, 10.5	78.6 (1.5)	7.2	3.5, 10.9
Cognitive functioning	83.6 (1.4)	83.1 (1.3)	−0.5	−4.0, 2.4	82.2 (1.3)	−1.4	−4.8, 1.5	83.8 (1.5)	0.2	−3.5, 3.1
Social functioning	73.8 (2.2)	72.2 (1.7)	−1.6	−6.7, 3.5	75.0 (1.8)	1.2	−4.2, 6.7	76.1 (2.0)	2.3	−2.8, 7.5
Fatigue	39.0 (2.1)	42.5 (1.7)	3.5	−1.2, 8.6	39.9 (1.8)	0.9	−3.3, 5.8	38.6 (2.1)	−0.4	−5.8, 4.4
Nausea and vomiting	16.2 (1.8)	18.8 (1.7)	2.6	−2.4, 7.6	13.7 (1.5)	−2.5	−6.7, 1.6	13.0 (1.9)	−3.2	−8.2, 1.7
Pain	32.7 (2.1)	24.4 (1.8)	−8.3	−13.2, −3.3	26.5 (2.0)	−6.2	−11.3, −1.2	25.8 (2.3)	−6.9	−12.5, −1.4
Dyspnea	10.3 (1.4)	13.6 (1.5)	3.3	−0.5, 7.0	12.1 (1.7)	1.8	−2.2, 5.7	14.3 (1.9)	4.0	−0.5, 8.5
Insomnia	35.6 (2.8)	23.8 (2.0)	**−11.8**	−18.0, −5.5	20.3 (2.0)	**−15.3**	−21.5, −9.1	22.6 (2.3)	**−13.0**	−19.3, −6.6
Appetite loss	35.2 (2.7)	33.4 (2.4)	−1.8	−8.5, 4.7	27.7 (2.2)	−7.5	−14.1, −1.1	25.9 (2.5)	−9.3	−15.2, −2.1
Constipation	21.2 (2.2)	14.2 (1.8)	−7.0	−12.2, −1.8	12.3 (1.7)	−8.9	−14.3, −3.6	13.0 (1.8)	−8.2	−12.8, −3.6
Diarrhea	15.5 (1.9)	26.4 (2.2)	**10.9**	5.6, 16.3	23.0 (2.1)	7.5	2.8, 12.4	17.6 (2.0)	2.1	−2.2, 6.4
Financial difficulties	10.5 (1.6)	11.0 (1.5)	0.5	−3.1, 4.2	10.5 (1.5)	0	−3.3, 3.4	10.4 (1.6)	−0.1	−3.3, 3.1
**QLQ-PAN26**										
Body image	25.0 (2.2)	24.1 (1.7)	−0.9	−5.8, 4.2	21.5 (1.7)	−3.5	−8.5, 1.6	23.8 (1.9)	−1.2	−6.5, 4.2
Pancreatic pain	39.3 (2.1)	23.1 (1.4)	**−16.2**	−21.0, −11.5	26.2 (2)	**−12.9**	−17.7, −8.2	26.2 (1.7)	**−13.1**	−17.7, −8.4
Eating related items	31.2 (2.4)	31.6 (2.1)	0.4	−5.6, 6.4	26.3 (2.2)	−4.9	−10.9, 1.1	26.9 (2.6)	−4.3	−10.5, 1.8
Altered bowel habit	28.2 (1.9)	32.9 (1.8)	4.7	−0.5, 9.8	34.8 (1.8)	6.6	1.9, 11.3	34.3 (2.2)	6.1	1.0, 11.1
Hepatic symptoms	22.3 (2.3)	9.3 (1.5)	**−13.0**	−18.1, −7.9	5.2 (0.9)	**−17.1**	−21.9, −12.4	7.2 (1.5)	**−15.1**	−20.2, −10.1
Health care satisfaction	35.9 (2.4)	39.2 (2.1)	3.3	−2.6, 9.3	43.8 (2.3)	7.9	1.8, 14.1	43.2 (2.8)	7.3	0.4, 14.3
Sexuality	51.0 (3.0)	52.6 (2.6)	1.6	−5.3, 8.4	53.8 (2.8)	2.8	−4.2, 9.8	52.7 (3.3)	1.7	−6.2, 9.5
Side effects	27.2 (1.9)	39.7 (1.7)	**12.5**	7.5, 17.4	34.9 (1.8)	7.7	2.8, 12.6	32.0 (2.1)	4.8	−0.2, 9.8
Cachexia	29.9 (1.9)	30.0 (1.7)	0.1	−4.7, 5.1	27.7 (1.8)	−2.2	−6.9, 2.5	24.3 (2.1)	−5.6	−10.7, −0.5
Indigestion	29.6 (2.3)	30.2 (2.0)	0.6	−5.2, 6.4	30.5 (2.1)	0.9	−4.9, 6.5	26.9 (2.5)	−2.7	−8.6, 3.1
Ascites	34.2 (2.3)	26.2 (2.0)	−8.0	−13.4, −2.6	29.1 (2.2)	−5.1	−10.6, 0.4	28.0 (2.6)	−6.2	−12.4, −0.1
Flatulence	33.6 (2.3)	39.8 (2.0)	6.2	0.5, 11.8	37.1 (2.1)	3.5	−2.0, 9.0	37.2 (2.4)	3.6	−2.0, 9.2
Fear of future health	63.9 (2.3)	53.5 (2.0)	**−10.4**	−15.7, −5.0	52.3 (2.2)	**−11.6**	−17.1, −6.1	50.2 (2.6)	**−13.7**	−19.8, −7.5
Ability to plan the future	42.5 (2.8)	43.5 (2.1)	1.0	−5.4, 7.6	39.7 (2.4)	−2.8	−9.5, 4.1	35.5 (2.8)	−7.0	−14.2, 0.2

Linear mixed model analysis at baseline (T0), during treatment (T1), 0–3 months after treatment (T2), and 3–6 months after treatment (T3). The delta (Δ) indicates the mean difference between the score at that time point compared to baseline value. SE, standard error; 95% CI, 95% confidence interval; n, number of patients who answered the specific question in the questionnaires. Higher mean scores indicate better quality of life for the functional scales, or more symptomatology (poorer quality of life) for the symptom scales.

Bold numbers indicate a clinically relevant difference.

Among the PAN26 subscales, pancreatic pain decreased clinically relevantly at T1 (MD: −16.2, 95% CI [−21.0, −11.5]), at T2 (MD: −12.9, 95% CI [−17.7, −8.2]), and T3 (MD: −13.1, 95% CI [−17.7, −8.4]). Hepatic symptoms also decreased clinically relevantly at T1 (MD: −13.0, 95% CI [−18.1, −7.9]), T2 (MD: −17.1, 95% CI [−21.9, −12.4]), and T3 (MD: −15.1, 95% CI [−20.2, −10.1]). Side effects increased clinically relevantly at T1 (MD: 12.5, 95% CI [7.5, 17.4]). Fear or future health deteriorated clinically relevantly at T1 (MD: −10.4, 95% CI [−15.7, −5.0]), T2 (MD: −11.6, 95% CI [−17.1, −6.1]), and T3 (MD: −13.7, 95% CI [−19.8, −7.5]).

### HRQoL of patients with metastatic PAC who received chemotherapy

In the M1-C-PAC group (Table 4), the mean GHS at baseline was 66.0 (SE: 1.7) and no clinically relevant changes were observed over time (Fig. 2). Of the QLQ-C30, pain was decreased clinically relevantly at T1 (MD: −10.8, 95% CI [−15.8, −5.8]). Also insomnia decreased clinically relevantly at T1 (MD: −10.4, 95% CI [−16.1, −4.8]) and T3 (MD: −10.6, 95% CI [−17.0, −4.1]). Additionally, constipation decreased clinically relevantly at T2 (MD: −10.5, 95% CI [−16.1, −4.7]).

**Table 4:** EORTC-QLQ-C30 and PAN26 subscales of patients with metastatic PAC who received chemotherapy (M1-C-PAC).

	T0 | Before treatment (n = 148)	T1 | During treatment (n = 215)			T2 | 0–3 months after treatment (n = 170)			T3 | 3–6 months after treatment (n = 95)		
	Mean (SE)	Mean (SE)	Δ	95% CI	Mean (SE)	Δ	95% CI	Mean (SE)	Δ	95% CI
**QLQ-C30**										
Global health status	66.0 (1.7)	64.9 (1.3)	−1.1	−5.1, 2.9	66.0 (1.4)	0	−4.3, 4.3	61.7 (2.1)	−4.3	−9.7, 1.0
Physical functioning	79.6 (1.6)	74.3 (1.3)	−5.3	−8.8, −1.7	73.1 (1.5)	−6.5	−10.2, −2.7	70.9 (1.9)	−8.7	−13.1, −4.3
Role functioning	66.9 (2.3)	61.4 (1.9)	−5.5	−11.0, −0.1	63.7 (2.1)	−3.2	−8.9, 2.5	65.4 (2.8)	−1.5	−8.2, 5.3
Emotional functioning	70.3 (1.6)	76.4 (1.4)	6.1	2.3, 9.8	77.0 (1.5)	6.7	2.9, 10.4	75.5 (1.8)	5.2	1.0, 9.3
Cognitive functioning	83.1 (1.5)	82.2 (1.4)	−0.9	−4.5, 2.7	82.0 (1.5)	−1.1	−5.1, 2.8	78.0 (1.9)	−5.1	−9.6, −0.7
Social functioning	73.0 (2.2)	69.7 (1.7)	−3.3	−8.2, 1.6	74.4 (1.8)	1.4	−3.7, 6.6	72.5 (2.6)	−0.5	−6.9, 6.0
Fatigue	38.4 (2.0)	44.2 (1.6)	5.8	1.2, 10.5	41.2 (1.8)	2.8	−2.1, 7.6	40.8 (2.4)	2.4	−3.3, 8.2
Nausea and vomiting	13.1 (1.7)	14.9 (1.4)	1.8	−2.5, 6.1	12.0 (1.6)	−1.1	−5.6, 3.4	11.9 (2.1)	−1.2	−6.3, 4.0
Pain	34.4 (2.1)	23.6 (1.7)	**−10.8**	−15.8, −5.8	28.6 (1.9)	−5.8	−11.1, −0.6	30.5 (2.5)	−3.9	−10.2, 2.2
Dyspnea	13.8 (1.9)	15.8 (1.6)	2.0	−2.4, 6.4	21.4 (1.8)	7.6	3.0, 12.2	20.1 (2.3)	6.3	0.9, 11.7
Insomnia	34.4 (2.3)	24.0 (2.0)	**−10.4**	−16.1, −4.8	24.9 (2.1)	−9.5	−15.0, −4.0	23.8 (2.7)	**−10.6**	−17.0, −4.1
Appetite loss	37.5 (2.5)	31.6 (2.1)	−5.9	−12.3, 0.5	28.0 (2.3)	−9.5	−16.1, −2.9	30.3 (3.0)	−7.2	−14.5, 0
Constipation	22.5 (2.4)	14.7 (1.7)	−7.8	−13.6, −2.1	12.1 (1.6)	**−10.4**	−16.1, −4.7	12.7 (2.3)	−9.8	−16.3, −3.4
Diarrhea	16.0 (2.1)	24.8 (1.7)	8.8	4.0, 13.7	18.5 (2.0)	2.5	−2.9, 7.9	19.0 (2.6)	3.0	−3.4, 9.5
Financial difficulties	6.4 (1.4)	9.0 (1.4)	2.6	−0.8, 5.9	8.4 (1.4)	2.0	−1.1, 5.1	6.1 (1.4)	−0.3	−3.6, 2.9
**QLQ-PAN26**										
Body image	24.2 (2.0)	26.6 (1.7)	2.4	−2.3, 7.2	28.3 (1.9)	4.1	−1.2, 9.4	23.5 (2.5)	−0.7	−7.1, 5.7
Pancreatic pain	38.8 (2.0)	23.3 (1.3)	**−15.5**	−20.0, −11.0	26.5 (1.7)	**−12.3**	−17.2, −7.4	29.8 (2.1)	−9.0	−14.4, −3.5
Eating related items	34.2 (2.4)	31.3 (2.0)	−2.9	−8.4, 2.6	27.2 (2.2)	−7.0	−13.1, −0.9	28.5 (2.9)	−5.7	−12.6, 1.0
Altered bowel habit	28.6 (1.7)	35.6 (1.7)	7.0	2.6, 11.4	32.4 (1.8)	3.8	−0.9, 8.4	28.7 (2.2)	0.1	−5.3, 5.5
Hepatic symptoms	15.6 (2.1)	4.6 (0.8)	**−11.0**	−15.3, −6.6	7.8 (1.0)	−7.8	−12.4, −3.2	7.3 (1.4)	−8.3	−13.4, −3.2
Health care satisfaction	35.0 (2.3)	36.5 (1.9)	1.5	−4.0, 7.1	36.0 (2.2)	1.0	−4.8, 7.0	36.9 (3.0)	1.9	−5.1, 9.0
Sexuality	49.7 (2.7)	60.4 (2.3)	**10.7**	4.6, 16.9	58.9 (2.6)	9.2	2.5, 16.0	55.0 (3.4)	5.3	−2.4, 13.1
Side effects	28.2 (2.0)	41.7 (1.6)	**13.5**	8.4, 18.4	35.3 (1.8)	7.1	1.9, 12.2	28.9 (2.4)	0.7	−5.2, 6.5
Cachexia	27.4 (1.9)	29.2 (1.6)	1.8	−2.5, 6.2	26.2 (1.8)	−1.2	−6.0, 3.7	25.1 (2.3)	−2.3	−7.7, 3.2
Indigestion	27.1 (2.7)	28.7 (1.9)	1.6	−3.6, 6.7	23.1 (2.2)	−4.0	−9.9, 1.8	26.8 (2.9)	−0.3	−7.3, 6.7
Ascites	38.3 (2.3)	27.2 (1.9)	**−11.1**	−16.6, −5.6	25.0 (2.2)	**−13.3**	−19.1, −7.5	26.8 (2.9)	**−11.5**	−18.4, −4.6
Flatulence	35.4 (2.4)	38.2 (2.0)	2.7	−2.8, 8.2	34.2 (2.2)	−1.2	−7.0, 4.6	37.6 (2.9)	2.2	−4.8, 9.1
Fear of future health	68.2 (2.3)	57.8 (1.9)	**−10.4**	−15.7, −5.0	60.9 (2.1)	−7.3	−12.7, −1.7	55.1 (2.7)	**−13.1**	−19.2, −7.0
Ability to plan the future	42.8 (2.7)	44.7 (2.1)	1.9	−4.4, 8.2	39.1 (2.2)	−3.7	−10.0, 2.6	28.1 (3.1)	−4.7	−12.4, 3.1

Linear mixed model analysis at baseline (T0), during treatment (T1), 0–3 months after treatment (T2), and 3–6 months after treatment (T3). The delta (Δ) indicates the mean difference between the score at that time point compared to baseline value. SE, standard error; 95% CI, 95% confidence interval; n, number of patients who answered the specific question in the questionnaires. Higher mean scores indicate better quality of life for the functional scales, or more symptomatology (poorer quality of life) for the symptom scales.

Bold numbers indicate a clinically relevant difference.

Of the PAN26 scales, pancreatic pain decreased clinically relevantly at T1 (MD: −15.5, 95% CI [−20.0, −11.0]) and T2 (MD: 12.3, 95% CI [−17.2, −7.4]). Ascites also decreased clinically relevantly at T1 (MD: −11.1, 95% CI [−16.6, −5.6]), T2 (MD: −13.3, 95% CI [−19.1, −7.5]), and T3 (MD: −11.5, 95% CI [−18.4, −4.6]). Hepatic symptoms decreased clinically relevantly at T1 (MD: −11.0, 95% CI [−15.3, −6.6]). Sexuality improved clinically relevantly at T1 (MD: 10.7, 95% CI [4.6, 16.9]). Side effects increased clinically relevantly at T1 (MD: 13.5, 95% CI [8.4, 18.4]). Fear of future health deteriorated clinically relevantly at T1 (MD: −10.4, 95% CI [−15.7, −5.0]) and T3 (MD: −13.1, 95% CI [−19.2, −7.0]).

### HRQoL of patients with PAC who received best supportive care

In the BSC group (Table 5), the mean GHS at baseline was 59.5 (SE: 1.9) and no clinically relevant changes were observed over time (Fig. 2). None of the other QLQ-C30 scales changed clinically relevantly either. Among the PAN26 scales, hepatic symptoms improved clinically relevantly 3–6 months after treatment (MD T1: −11.8, 95% CI [−19.4, −4.1]). Flatulence deteriorated clinically relevantly 3–6 months after diagnosis (MD T1: 13.6, 95% CI [6.2, 20.9]).

**Table 5:** EORTC-QLQ-C30 and PAN26 subscales of patients who received best supportive care (BSC).

	T0 | 0–3 months after diagnosis (n = 148)	T1 | 3–6 months after diagnosis (n = 56)		
	Mean (SE)	Mean (SE)	Δ	95% CI
**QLQ-C30**				
Global health status	59.5 (1.9)	61.4 (2.5)	1.9	−3.5, 7.5
Physical functioning	68.1 (2.1)	61.5 (2.8)	−6.6	−11.6, −1.5
Role functioning	60.3 (2.8)	54.3 (3.9)	−6.0	−13.4, 1.5
Emotional functioning	73.3 (2.0)	79.1 (2.1)	5.8	0.8, 10.6
Cognitive functioning	79.4 (1.9)	78.2 (2.5)	−1.2	−6.6, 4.2
Social functioning	71.0 (2.4)	70.1 (3.6)	−0.9	−8.3, 6.5
Fatigue	46.2 (2.5)	50.8 (3.6)	4.6	−2.3, 11.5
Nausea and vomiting	18.2 (2.0)	15.0 (2.4)	−3.2	−8.6, 2.2
Pain	34.3 (2.4)	35.7 (3.5)	2.4	−4.3, 9.0
Dyspnea	16.4 (3.1)	14.2 (3.1)	−1.8	−9.0, 5.2
Insomnia	31.8 (2.6)	28.8 (4.1)	−3.0	−11.9, 5.9
Appetite loss	43.6 (3.0)	37.6 (4.7)	−6.0	−16.3, 4.2
Constipation	24.6 (2.7)	20.0 (3.6)	−4.6	−13.3, 4.1
Diarrhea	18.2 (2.2)	14.3 (3.4)	−3.9	−11.0, 3.1
Financial difficulties	3.3 (1.0)	7.0 (2.0)	3.7	1.0, 6.4
**QLQ-PAN26**				
Body image	25.7 (2.1)	26.2 (3.2)	0.5	−6.1, 7.0
Pancreatic pain	31.9 (2.1)	28.7 (3.3)	−3.2	−10.1, 3.7
Eating related items	38.8 (2.8)	35.4 (4.4)	−3.4	−13.0, 6.3
Altered bowel habit	33.3 (2.1)	31.4 (3.2)	−1.9	−8.7, 4.8
Hepatic symptoms	22.1 (2.7)	10.3 (2.8)	**−11.8**	−19.4, −4.1
Health care satisfaction	30.9 (2.2)	31.4 (3.7)	0.5	−7.8, 8.9
Sexuality	53.8 (3.5)	55.5 (5.4)	1.7	−10.0, 13.4
Side effects	32.0 (2.0)	32.5 (3.3)	0.5	−6.8, 7.8
Cachexia	36.2 (2.2)	34.4 (3.3)	−1.8	−9.2, 5.6
Indigestion	33.7 (2.7)	31.0 (3.6)	−2.7	−11.2, 5.8
Ascites	32.4 (2.5)	32.6 (3.8)	0.2	−7.7, 8.0
Flatulence	36.9 (2.4)	50.5 (3.6)	**13.6**	6.2, 20.9
Fear of future health	64.0 (2.5)	54.1 (3.8)	−9.9	−17.7, −2.2
Ability to plan the future	47.4 (3.1)	47.3 (4.9)	−0.1	−10.6, 10.4

Linear mixed model analysis at baseline (T0), during treatment (T1), 0–3 months after treatment (T2), and 3–6 months after treatment (T3). The delta (Δ) indicates the mean difference between the score at that time point compared to baseline value. SE, standard error; 95% CI, 95% confidence interval; n, number of patients who answered the specific question in the questionnaires. Higher mean scores indicate better quality of life for the functional scales, or more symptomatology (poorer quality of life) for the symptom scales.

Bold numbers indicate a clinically relevant difference.

## Discussion

This nationwide, multicenter prospective longitudinal cohort study investigated HRQoL in patients with PAC undergoing various treatments or best supportive care. In none of the four patient groups were clinically relevant changes in GHS observed. Certain subscales of the QLQ-C30 and PAN26 questionnaire demonstrated improvements or deteriorations of a clinically relevant magnitude.

The improvement or deterioration observed in the subscales may be due to effects of complications or symptoms associated with PAC. Alternatively, the changes may be influenced by treatment effects, including therapeutic benefits or adverse treatment reactions. In the R-PAC group, most domains deteriorated (i.e., decrease in functioning score or increase in symptom score) during treatment, but returned to baseline level 3–6 months after treatment. This might be explained by an initial negative impact of surgery on HRQoL due its side effects or post-operative complications, and a subsequent positive impact on HRQoL by delaying progression, consistent with prior research.^5^, ^6^, ^7^, ^8^^,^^25^

In the two cohorts that received chemotherapy (C-PAC and M1-C-PAC), clinically relevant decreases in symptom scores were observed for insomnia, pancreatic pain, and hepatic symptoms. However, these patients also reported clinically relevant side effects during treatment. Our findings suggest that patients with non-metastatic or metastatic disease receiving chemotherapy (and the necessary placement of a stent) derive significant clinical benefit from their treatment. This primarily manifests through the alleviation of disease-related symptoms and reduction of concerns, thereby potentially maintaining their HRQoL. For example, pancreatic pain may decrease due to tumor load reduction, while hepatic symptoms (jaundice) may improve through the alleviation of mechanical obstruction.

This potential clinical benefit of treatment with chemotherapy is consistent with previous literature suggesting that early palliative care may significantly improve both HRQoL and mood in patients with cancer in general and PAC in particular.^13^^,^^26^, ^27^, ^28^, ^29^, ^30^ This is important, as palliation rather than curation remains the most relevant treatment goal in the great majority of patients with PAC. However, despite advantages of chemotherapy, it can cause common toxicities such as vomiting and nausea, diarrhea, fatigue, loss of appetite and neuropathy, which may impact HRQoL adversely.^31^^,^^32^ This was observed in both the C-PAC and M1-C-PAC group, where complaints of diarrhea and side effects increased clinically relevantly during treatment, potentially attributable to the effects of chemotherapy.

Across all aforementioned treatment groups (R-PAC, C-PAC, M1-C-PAC), there was a clinically relevantly increase in fear of future health. This rise in concerns may stem from fear of progression or recurrence (FOP). A Dutch study reported that over half of patients with PAC had high worry about progression.^33^ In our study, patients in the R-PAC group had a lower baseline score than patients in the M-PAC and M1-PAC group. This difference may be attributed to surgical resection being a treatment with curative intent, while palliative chemotherapy may prolong life, but cure cannot be achieved. The longitudinal increase of fear of future health may be explained by worsening of prognosis due to an advanced disease stage.

Notably, the BSC group demonstrated predominantly stable HRQoL outcomes. This is in contrast with prior studies that described significant impairments in all domains of HRQoL among patients with advanced cancer who received BSC, particularly in the final months of life.^29^^,^^34^, ^35^, ^36^, ^37^ However, the progressively increasing mortality rates in the BSC group reflect disease severity and poor treatment outcomes, underscoring the challenges in capturing longitudinal HRQoL data in this population. These high mortality rates contribute to selection bias, as patients who survive longer and are in better clinical condition are more likely to complete questionnaires. Consequently, the HRQoL results at later time points may disproportionally represent patients with more favorable prognoses.

The variation in return rates across treatment groups further highlights the influence of selection bias. Return rates were relatively low at T0, particularly in the R-PAC group, possibly due to challenges faced shortly after diagnosis or early interventions. While return rates improved at later time points, these data likely reflect patients in more stable conditions. Consequently, it is important to consider these patterns when interpreting HRQoL outcomes, as the data may over-represent healthier patients. Additionally, survival plays a critical role in shaping the study population over time. Mortality not only limits data availability but also alters the characteristics of the remaining participants, further influencing observed HRQoL trends.

The current study has several limitations. First, we used a 10-point change to determine clinical relevance, as proposed by Osoba.^22^ A recent study by Musoro et al. investigated the minimally important difference or change (MID) for the QLQ-C30 questionnaire, based on a number of cancer clinical trials across nine different cancer types.^38^ They found that no single MID applies to all QLQ-C30 scales and cancer types. However, MIDs were found to range from 5 to 10 points across most scales. Unfortunately, PAC was not among the tumor types they investigated. We therefore adopted the more stringent 10 points difference that Osoba advocated to avoid over-interpretation of the results. Second, while mortality data were available, no data were available for patients who were lost to follow-up due to disease progression. Given the poor survival in PAC, attrition likely resulted from either disease progression or mortality. This aligns with the progressively increasing mortality rates observed in our study, particularly in the BSC group. Such dropout is especially concerning for endpoints that may be directly related to the measured outcome. Recent advancements in statistical methods, such as the estimand framework, which can involve data imputation, have been developed to address missing data and better align analyses with clinical objectives, thus improving the quality and interpretability of patient-reported outcomes.^39^ Since our statistical model could only take ‘missing data at random’ into account, it cannot fully account for missing data due to disease progression or mortality. Consequently, the results are only generalizable to those patients who are able to complete HRQoL questionnaires and not to those lost to follow-up due to disease progression or mortality. However, our aim was to evaluate the HRQoL in the clinical setting, focusing on patients who have undergone surgical treatment, systemic treatment or BSC. Imputing lower scores for patients lost to follow-up could introduce bias by artificially lowering overall scores and does not align with clinical practice, as deceased patients are no longer part of the healthcare setting. Third, we performed an extensive number of statistical tests, which increased the likelihood of finding statistically significant results by chance. However, by focusing solely on clinically relevant findings, we aimed to strengthen the robustness and practical relevance of our conclusions. Fourth, the timing of the T1 assessment varied between patients due to individual treatment schedules. For patients who completed multiple questionnaires during treatment, one was randomly selected to represent T1, which may introduce variability in interpreting HRQoL changes. Fixed time points in future studies could improve consistency. Furthermore, we did not stratify for the type of chemotherapy administered, which could influence HRQoL. Additionally, HRQoL may be influenced by various factors throughout the disease course, such as age, performance status and comorbidities. In our analysis, we did not adjust for these factors, as the focus was not on direct group comparisons but on preserving the natural variability within treatment groups and exploring longitudinal HRQoL changes. However, this approach may influence the interpretation of trends over time. Lastly, the relatively young median age of patients with metastatic PAC who received chemotherapy likely contributed to the high use of FOLFIFIRNOX. As a result, our findings may not fully represent older metastatic PAC patients, who are more often treated with gemcitabine based regimens. Therefore, caution is needed when generalizing our results to older populations. Regarding strengths, this is the first-ever population-based longitudinal study to assess HRQoL in patients with localized, locally advanced, and metastatic PAC undergoing various treatment regimens. Additionally, the non-selective inclusion of this study enhances its external validity.

In conclusion, this nationwide study has provided novel insights into the longitudinal HRQoL of patients with PAC up to 6 months after treatment or diagnosis, including various treatment regimens or BSC. Different treatment strategies demonstrated both positive and negative impacts on several HRQoL domains. These findings can facilitate SDM by enabling healthcare providers to counsel individual patients regarding the potential effect of a treatment strategy on their HRQoL.

## Contributors

AMG: Study concept and design, data access and verification, data analysis and biostatistics, writing—original draft, and writing—review & editing; PAJV: Study concept and design, data access and verification, and writing—review & editing; NMF: Data access and verification, data analysis and biostatistics, writing—original draft, and writing—review & editing; JWW and HWMvL: Study concept and design, writing—review & editing and supervision; MAGS, ENP, LGvdG, GAC, JdVG, MYVH, GJC, MWJS, LAD, and MGB: Writing—review & editing. Pancreatic Cancer Project, Netherlands Cancer Registry: data acquisition. All authors read and approved the final version of the manuscript.

## Data sharing statement

The data used in this study are available upon reasonable request and are subject to a data sharing agreement.

## Declaration of interests

JDV has served as a consultant for Amgen, AstraZeneca, MSD, Pierre Fabre, and Servier, and has received institutional research funding from Servier. All outside the submitted work. HWW has served as a consultant for AstraZeneca, MSD and Servier, and has received institutional research funding from Servier, MSD and Nordic. HWMvL declares research funding and/or medication supply from Bristol-Myers Squibb, Bayer Schering Pharma, Celgene, Janssen-Cilag, Lilly, Nordic Pharma, Philips Healthcare, Roche, Merck Sharp and Dohme, Servier, Incyte, and consultant/advisory board members for Lilly, Nordic Pharma, Bristol-Myers Squibb, Dragonfly, Merck Sharp and Dohme, Servier, outside the submitted work.
